# An online survey on burden of illness among families with post-stem cell transplant mucopolysaccharidosis type I children in the United States

**DOI:** 10.1186/s13023-019-1027-3

**Published:** 2019-02-18

**Authors:** Therese Conner, Francesca Cook, Vivian Fernandez, Karen Rascati, Vanessa Rangel-Miller

**Affiliations:** 1 REGENXBIO Inc., 9600 Blackwell Road, Suite 210, Rockville, MD 20850 USA; 2 University of Texas at Austin, College of Pharmacy, Austin, TX USA; 3 Invitae, San Francisco, CA USA

**Keywords:** Mucopolysaccharidosis type I, Family burden of illness, Hurler syndrome

## Abstract

**Background:**

Severe mucopolysaccharidosis type I (also known as Hurler syndrome) is a rare devasting recessive genetic disease caused by the deficiency of an enzyme. Hematopoietic stem cell transplant is the standard of care in the United States, usually conducted before the child is 3 years of age, but little is known about the continued medical and educational needs of the child after transplant. A greater understanding of the burden of illness on the primary caregiver is also needed. Therefore, this online survey sought to gather information on the burden of severe MPS I in the United States at least 1 year after transplant.

**Results:**

Thirty-two respondents reported that children with severe MPS I have significant medical and educational needs after transplant. Healthcare resource use was frequent, especially in the outpatient setting specifically for bone, cardiac, and vision complications that were not relieved by HSCT. Twenty-five percent of the children had been hospitalized at least once in the last year and two had been hospitalized twice. The most common reasons for overnight hospitalizations included orthopedic surgeries and respiratory infections. Among children ages 5 and older, only 3 of 28 (11%) were able to attend school with no special support. While caregivers were generally satisfied with the healthcare services their child receives, 69% of working caregivers reported negative impact on their ability to conduct work tasks, and 54% of caregivers did not work so that they could care for the child.

**Conclusions:**

Results suggest that severe MPS I children continue to  require medical care and special support for education. Future research on the burden of illness on families affected by severe MPS I is needed to better understand total cost of care, and to identify therapies and interventions that reduce burden of illness. Future studies that compare cost of and access to health care in different countries may provide a more global view of the burden of MPS I.

## Introduction

Mucopolysaccharidosis (MPS) type I is a rare and devastating recessive genetic disease with an estimated prevalence of 2.8 in 100,000 live births in the United States (US) according to birth screening data [1]. The disease is caused by the deficiency of α-L-iduronidase (IDUA), an enzyme required for the lysosomal catabolism of the ubiquitous complex polysaccharides [2]. These polysaccharides, called glycosaminoglycans (GAGs), accumulate in tissues, resulting in characteristic storage lesions and diverse disease sequelae. Patients may exhibit multiple symptoms, including short stature, bone and joint deformities, coarsened facial features, hepatosplenomegaly, cardiac valve disease, obstructive sleep apnea, recurrent upper respiratory tract infections, hearing impairment, carpal tunnel syndrome, and vision impairment due to corneal clouding [3]. In addition, many patients develop symptoms related to GAG storage in the central nervous system (CNS), including hydrocephalus, spinal cord compression, and cognitive impairment [2–4].

Patients with MPS I exhibit a broad spectrum of disease severity and extent of CNS involvement. Hurler syndrome, or severe MPS I, typically involves significant developmental delay and cognitive decline starting shortly after birth and progressing rapidly [3, 5, 6]. Untreated Hurler children would likely die in the first decade of life, but two treatments that slow progression and provide benefits, hematopoietic stem cell transplant (HSCT) and enzyme replacement therapy (ERT), are available in the US. HSCT has been shown to increase survival beyond the third decade of life and to decrease incidence of cardiac mortality. Outcomes are maximized when HSCT is performed before the age of 2.5 years and within 6 months of diagnosis [2, 7–11]. One year after successful HSCT, improvements in hepatosplenomegaly, characteristic facial phenotypes, joint stiffness, sleep apnea, cardiac disease (including myocardial function and permeability of coronaries), and hearing loss have been reported [2, 9, 10, 12]. However, HSCT carries risk of mortality and morbidity, and does not prevent progression of some disease-associated sequelae such as thickening of cardiac valves [2, 8, 10, 12–15]. Furthermore, orthopedic consequences of the disease will require surgical intervention in the majority of post-transplant patients as HSCT has little impact on bone disease [2, 9, 16]. Finally, cognitive decline that occurred prior to transplant will likely not be reversed [15].

ERT with laronidase as a weekly intravenous (IV) infusion is another treatment option for MPS I patients. Benefits of ERT include improved pulmonary function, improved walking capacity, reduced hepatosplenomegaly, and clinical changes with improved joint range of motion, decreased pain, and improved activity [2, 17–20]. However, since IV laronidase does not cross the blood-brain barrier, it does not treat the CNS manifestations of MPS I, and thus patients may experience neurocognitive deficits throughout life.

Given that the consequences of severe MPS I are chronic and debilitating, it is expected that families with MPS I children would experience significant economic, social, and humanistic burdens. Healthcare utilization, educational needs for the child, and caregiver burden in the US have not been well-documented in the literature. A few non-US studies have assessed the burden of a variety of MPS types and other similar lysosomal storage diseases with regard to healthcare use, cost of care, caregiver time, and impact on work and family life. An Australian study on multiple metabolic disorders conducted a survey among 30 families to assess the health, psychosocial, and financial impact of caring for a child with a rare disease [21]. The researchers reported that in a 12-month period, the 30 children had 168 visits to general practitioners and 260 visits to specialists. Seventy percent of children had at least one hospital admission, including one patient who had 16 admissions in 12 months. Most families (77%) received financial assistance, but half of those families believed it was insufficient.

Another study assessed healthcare utilization in 51 MPS type II patients in France, primarily (69.2%) those with severe subtype [22]. Among respondents, 51% had been admitted to hospital in the last 12 months because of the disease, with a mean length of stay of 10.4 days (range 1–120 days), and 25.5% had been to the emergency room a mean number of 2 times, primarily for respiratory symptoms. Medical specialists most often seen over the last 12 months included dentists (43%), ear, nose and throat specialists (49%), and ophthalmologists (29.4%). Eighty-four percent had seen a physical therapist an average of 7.4 times/year and 40% had seen a speech therapist an average of 5.3 times/year. Of the respondents, 72.5% reported they used social services assistance for help with home care aides and financial assistance. Among the 39 families that received educational financial support, 16 received no other aid while 13 also received disability living allowances. Only 3 families received no financial aid.

Aldenhoven and researchers assessed quality of life in 63 Hurler patients, as reported by parents, including functional and psychosocial health and perception of care in one of seven European transplant centers [23]. Among these transplanted patients, 46% had cognitive development impairment and 51% had growth retardation. The researchers found significantly reduced functional health scores among the children, compared to normative data, while psychosocial health appeared similar to normative data. Parents were satisfied overall with the healthcare they received from transplant centers, with respect to promoting partnerships, providing general and specific information, providing coordinated and comprehensive care, and providing respectful and supportive care.

Pentek et al. assessed disease burden in 120 patients (74 children and 46 adults) and 66 caregivers among the MPS community, across many disease types, in seven European countries (Italy, Spain, Germany, France, Hungary, Sweden, and Bulgaria) as part of a project referred to as BURQOL-RD (https://www.eurordis.org/content/burqol-rd-project-0) [24]. Multiple online questionnaires were provided in these countries, including questionnaires for patient completion and for the patients’ main informal caregiver. A total of 101 (92%) of patients said they needed help from a caregiver to perform every day activities. Mean care time for children was 51.0 (std = 64.4) hours per week and did not differ compared to time caring for adult patients (mean time 51.8 h, std 68.2). Only 20% of 46 adult patients were employed, and 35% were on disability pension.

The purpose of this study was to gain a better understanding of the burden, including healthcare resource use, educational support needs and work productivity, among families with children with severe MPS I in the US. There is a paucity of data available in the literature in the US assessing family burden and this study sought to contribute to current knowledge, identify existing unmet needs and areas for further study, and provide a mechanism to share our results with survey respondents and the MPS I community.

## Methods

Parents living in the US with children with severe MPS I who were younger than 18 years of age were invited to participate in December 2017 and January 2018 via ConnectMPS, an advocacy-supported patient registry of MPS families. Invitae, the parent company of ConnectMPS, recruited its members through the registry and the National MPS Society, a patient advocacy organization, promoted awareness through outreach to their members. Honoraria were given to families who completed the survey, distributed by Invitae so that the study sponsor remained blinded to respondent identity. An Institutional Review Board approved the study, and parents were required to provide online consent prior to completing the survey. No patient nor family identifiers were gathered in the survey itself so that respondents remained anonymous to the survey sponsor.

The online survey was modified from the BURQOL-RD project [24] to be relevant for US residents. The survey was made available through the ConnectMPS registry platform so that only registered members had access, and it took less than an hour to complete.

## Results

A total of 32 parents of post-HSCT severe MPS I children living in the US completed the survey. The children were diagnosed with MPS I at 3 years or younger (3 missing responses). All 32 children were transplanted at 3 years of age or younger. Twenty-five (78%) of the children qualified as disabled under Medicare/Social Security, and 80% of those children received monthly Social Security/Disability Living Benefits. Ninety percent received $750 or less per month. Other demographics are shown in Table 1.

**Table 1:** Child Demographics and Medical History (*N* = 32)

Description	
Current Age, Years - mean (std)	9.4 (4.9)
Gender	
Female – n (%)	17 (53)
Male – n (%)	15 (47)
US Region of Residence – n (%)	
South	16 (50)
Northeast	7 (22)
West	6 (19)
Midwest	3 (9)
Total Members in Household – mean (range)	4 (2–7)
Healthcare Insurance Plan^a^ – n (%)	
Private/Commercial Only	19 (61)
Medicaid/CHIP Only	3 (10)
Private + Medicaid	6 (19)
Other or Combinations	3 (10)

^a^1 missing

### Educational needs

Parents were asked to describe their child’s school status. Among children ages 5 and older, only 3 of 28 (11%) were able to attend school with no special support. Fifteen (54%) required personnel support, 9 (32%) attended a special school/program, and 1 (4%) was home schooled/received private lessons.

### Healthcare services

Parents were asked to report on their child’s recent enzyme replacement therapy (ERT) use in the previous month. Fourteen children (44%) had received IV laronidase in the prior month, according to parent report. The survey gathered frequency of physician visits and other outpatient healthcare service use over the prior 6 months; these visits were doubled to estimate annual use per patient. Parents were asked to recall how many pediatrician/family physician outpatient visits the child had incurred in the last 6 months. Twenty-four children had incurred one or more visits in the last 6 months. The number of visits ranged from 0 to 35, with a median of three visits (mean of 6.7 and standard deviation of 7.7) in the last 6 months.

Similarly, parents were asked to recall frequency of physician specialist visits. Table 2 shows estimated frequency per patient per year (PPPY) and the percent of those with complete, partial, or no insurance coverage to pay for the visits. Parent recall of other ancillary services are shown in Table 3. Table 4 shows the frequency of various tests and exams over the last 6 months as well as estimated visits PPPY and insurance coverage for those tests.

**Table 2:** Frequency of Physician Specialist Visits and Insurance Coverage

Description	N Children with 1+ visit	Total Visits in Last 6 Months	Estimated PPPY	Insurance Coverage per Child n (%)^a^		
				Complete	Partial	None
Cardiologist	26	43	2.69	21 (81)	5 (19)	–
Dermatologist	15 ^b^	22	1.38	11 (79)	2 (14)	1 (7)
Endocrinologist	22	32	2.0	17 (77)	5 (23)	–
Gastroenterologist	9	13	0.81	8 (89)	1 (11)	–
General surgeon	10	16	1.0	7 (70)	3 (30)	–
Hematologist	16 ^b^	35	2.19	13 (87)	2 (13)	–
Immunologist	8	23	1.44	7 (88)	1 (12)	–
Nephrologist	12	25	1.56	10 (83)	1 (8)	1 (8)
Neurologist	20 ^b^	29	1.81	15 (79)	4 (21)	–
Neurosurgeon	12 ^c^	19	1.19	9 (90)	1 (10)	–
Oncologist	10	20	1.25	7 (70)	3 (30)	–
Ophthalmologist	21 ^c^	39	2.44	14 (74)	5 (26)	–
Orthopedist	25 ^d^	55	3.44	14 (74)	5 (26)	–
Otolaryngologist	15^b^	24	1.50	11 (79)	3 (21)	–
Psychiatrist	7	16	1.0	7 (100)	–	–
Pulmonologist	18^b^	26	1.63	12 (71)	4 (24)	1 (6)
Rheumatologist	6	17	1.06	6 (100)	–	–
Traumatologist	6	13	0.81	5 (83)	1 (17)	–
Urologist	11	30	1.88	10 (91)	1 (9)	–

*PPPY* per patient per year

^a^may not equal 100% due to rounding; ^b^1 missing; ^c^2 missing; ^d^6 missing

**Table 3:** Frequency of other specialist visits and insurance coverage

Description	N Children 1 + visit	Total Visits in Last 6 Months	Estimated PPPY	Insurance Coverage per Child n (%)^a^		
				Complete	Partial	None
Behavioral therapist	19 ^b^	63	3.94	17 (100)	–	–
Chiropodist	10	21	1.31	9 (90)	1 (10)	–
Dentist	25 ^c^	39	2.44	19 (79)	5 (21)	–
Genetic counselor	9	13	0.81	7 (78)	2 (22)	–
Occupational therapist	22	341	21.31	19 (86)	3 (14)	–
Optometrist	13 ^b^	24	1.50	9 (82)	1 (9)	1 (9)
Physical therapist	25	419	26.19	19 (76)	6 (24)	–
Psychologist	8	24	1.50	7 (88)	1 (12)	–
Speech therapist	19	291	18.19	17 (89)	1 (5)	1 (5)

*PPPY* per patient per year

^a^percentages may not equal 100% due to rounding; ^b^2 missing; ^c^1 missing

**Table 4:** Frequency of Medical Tests and Examinations and Insurance Coverage

Description	N Children 1+ visit	Total Visits in Last 6 Months	Estimated PPPY	Insurance Coverage Per Child n (%)^a^		
				Complete	Partial	None
Audiometry	23	42	2.6	18 (78)	5 (22)	–
Blood Tests	31	90	5.6	24 (77)	7 (23)	–
Bronchoscopy	10	21	1.3	8 (80)	2 (20)	–
Echocardiogram	27	40	2.5	20 (74)	7 (26)	–
Electrocardiogram	27	47	2.9	21 (78)	6 (22)	–
Electromyogram	16	24	1.5	13 (81)	3 (19)	–
Lumbar puncture	11	23	1.4	8 (73)	3 (27)	–
Radiology (X-ray)	28	73	4.6	23 (82)	5 (18)	–
MRI or CT	25	40	2.5	21 (84)	3 (12)	1 (4)
Slit lamp	22	37	2.3	16 (73)	5 (23)	1 (5)
Spirometry	20	45	2.8	15 (75)	5 (25)	–
Urinalysis	23^a^	47	2.9	17 (77)	5 (23)	–

MRI or CT magnetic resonance imaging or computerized tomography, PPPY per patient per year

^a^may not equal 100% due to rounding

The survey also gathered information on the frequency of emergency room/urgent care visits in the previous 6 months. Twelve children (38%) had sought urgent or emergency care at least once (range of 1 to 4 visits) in the last 6 months. Parents were asked to report day surgeries and overnight hospitalizations in the previous 12 months. Eight (25%) children had one or more day surgery in the prior year. The most common reasons included removal/replacement of medical devices, orthopedic procedures, and interventional cardiac procedures. Eight (25%) of the children had been hospitalized at least once in the last year and two had been hospitalized twice. The most common reasons for overnight hospitalizations included orthopedic surgeries and respiratory infections.

Parents were asked to rate their satisfaction at the time of survey with the healthcare services their child received, using a numeric scale of 1 to 7, indicating very unsatisfied (1) to very satisfied (7). Responses are shown in Table 5. Twenty-seven parents (84%) reported feeling satisfied to some degree with the healthcare services their child received.

**Table 5:** Parent Satisfaction with Healthcare Services (*n* = 32)

	Very Unsatisfied	Mostly Unsatisfied	Somewhat Unsatisfied	Neither unsatisfied /satisfied	Somewhat Satisfied	Mostly Satisfied	Very Satisfied
n	0	3	1	1	10	7	10
%^a^	–	9	3	3	31	22	31

^a^may not equal 100% due to rounding

### Caregiver burden

Twenty-eight (88%) parents identified themselves as the primary caregiver of their child. One parent reported that her spouse was the primary caregiver, and three parents reported that they had a professional caregiver. If the parent completing the survey was also the primary caregiver, the survey presented a series of questions about caregiving. If the parent completing the survey was not the primary caregiver, they did not answer the caregiver questions. Among those responding, seven were employed part time, six were employed full time, and 15 were stay-at-home parents (*n* = 14) or retired/on a pension (*n* = 1). Three (23%) of the 13 employed parental primary caregivers reported that they had reduced their work hours per week over the last 6 months and nine (69%) stated they had difficulty performing work tasks in the last 6 months. Of the 28 caregiving parents, eleven (39%) reported taking time off from work in the last 6 months to care for their child; six of the eleven (55%) had taken 7 or more days off.

The survey requested an estimation of the number of hours per day the primary caregiver spent on activities for their child. The results are shown in Table 6. Notably hours spent caring for older children were similar to the overall cohort.

**Table 6:** Caregiver Hours^a^ Spent on Daily Activities for Child

Daily Activities	n	Mean	St Dev	Median	Min	Max
On basic hygiene, dressing or changing the child	26	1.34	0.74	1	0	3
Subset: children ages 11–17 yrs	8	1.34	0.77	1.5	0	2
On feeding the child	26	1.20	1.18	1	0	6
Subset: children ages 11–17 yrs	8	1.41	1.89	1	0	6
On helping the child to move	26	1.55	0.97	2	0	4
Subset: children ages 11–17 yrs	8	1.34	0.93	1	0	3
On cooking and preparing special meals	26	2.16	2.99	1	0	12
Subset: children ages 11–17 yrs	8	2.06	3.3	1	0	10
On administering drugs/treatments	26	1.51	1.01	1	0	4
Subset: children ages 11–17 yrs	8	1.75	0.89	1.5	1	3
On bathing or showering the child	26	0.82	0.65	1	0	2
Subset: children ages 11–17 yrs	8	0.75	0.64	0.75	0	2
TOTAL Hours Per Day	26	8.58	5.98	7.5	1	25
Subset: children ages 11–17 yrs	8	8.66	7.28	7.13	1	25

^a^total may be greater than 24 h due to rounding and multiple tasks spent in same hours; 2 parents’ responses missing

## Discussion

Several studies have described the continued needs of children with MPS I with regard to healthcare and educational services. Functional status of children may improve in some ways after HSCT, especially when HSCT is conducted as early in age as possible [25], but multiple studies have reported that children experience life-long health problems and cognitive challenges. In Australia and France, studies on metabolic disorders and MPS II reported that children required frequent visits to specialists and incurred costly hospitalizations [21, 22]. The findings from this study suggest that children with severe MPS I in the US experience similar needs.

Healthcare needs reported in this study were similar to those in the non-US studies. US parents of MPS I children in this study reported frequent visits to healthcare providers, especially to physical therapists (26.2 visits PPPY), occupational therapists (21.3 visits PPPY), and speech therapists (18.2 visits PPPY), indicating continuous impairment in physical function and likely impacting psychosocial function. Frequent visits to orthopedists (3.44 visits PPPY), cardiologists (2.69 PPPY), and ophthalmologists (2.44 PPPY) suggest that, as supported by previous research, children experience bone, cardiac, and vision complications that are not relieved by HSCT. An unexpected finding was that 44% of parents reported their child had received ERT in the previous 30 days. This warrants further investigation, as current medical evidence does not support the extended use of ERT after transplant. However, studies of long-term use of ERT post-transplant may have enrolled children who were also captured through this survey.

Parents reported overall satisfaction with the healthcare services they received, similar to Aldenhoven et al.’s report from multiple European transplant centers [23]. Unlike the study by Aldenhoven, however, this study did not gather clinical data such as physical and neurocognitive functional status.

Furthermore, this US survey found that almost 90% of school-age MPS I children attended school with special support (or were home-schooled), which may indicate that these children were neurocognitively impaired, were not within normal child development ranges, and/or had other challenges that required special support. According to Shapiro et al., 59% of severe MPS I children are stable but remain cognitively impaired after transplant [15].

There are advantages and disadvantages to conducting on-line surveys. Resources needed for distribution and administration are lessened (in comparison to oral interviews or paper surveys), and parents could start and stop the online survey as their time permitted. Skip logic and multiple choice/drop down menu choices may have minimized overall time spent, and may have reduced misunderstanding of completion instructions as well. However, this online survey may have had limitations as well; for example, some items on the survey appeared to be skipped, resulting in missing data. The survey did not include options for adding free-text comments or additional information other than the questions being asked. Parents were asked to recall healthcare resource use from as recently as a month prior to as long as 12 months prior. Finally, this online survey afforded no clinical verification that the children had a confirmed diagnosis of severe MPS I, or that the respondent was indeed a parent or legal guardian of the child.

## Conclusion

This is one of the first studies conducted in the US on the burden of illness in severe MPS I. The high response rate from families underscores MPS I family interest in the topics covered in the survey, indicating further research and development of caregiver-based burden of illness tools is warranted. These tools combined with related health economics and outcomes (HEOR) studies would enable further exploration of the economic impact of the disease on families and society, the overall burden of disease over time, and further assessment of caregiver time needed for older MPS I children and young adults. Incorporating burden of illness tools and HEOR assessments into MPS I clinical development programs has the potential to provide meaningful data on which to assess the value of new novel therapies to families and health systems as well as assist drug developers, insurers and educational systems to design improved patient-focused support services. Future research might compare access to and cost of health care in multiple countries to better understand the global impact of MPS I. Other potentially valuable studies might assess the impact of early HSCT on neurocognitive function and the degree to which needs for healthcare services and education might differ. In conclusion, while medical advances have prolonged life and reduced morbidity in the severe MPS I population, this study shows that significant medical and educational needs remain, prompting the need for novel therapies that lessen the burden of illness.
